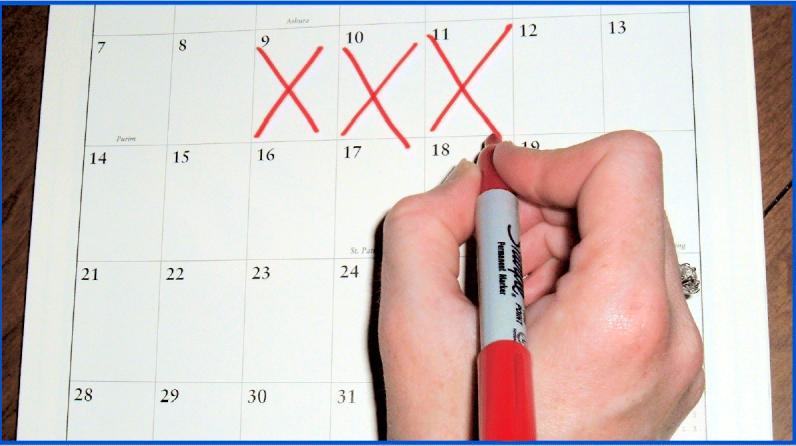# Headliners: Reproductive Health: Effects of Organochlorine Compounds on Menstrual Cycles

**Published:** 2005-07

**Authors:** Jerry Phelps

Windham GC, Lee D, Mitchell P, Anderson M, Petreas M, Lasley B. 2005. Exposure to organochlorine compounds and effects on ovarian function. Epidemiology 16:182–190.

Over the past 20–30 years, environmental health scientists have expressed increasing concern about endocrine disruptors, chemicals that appear to disrupt hormonal activity in humans and animals. Research has shown that women exposed at various life stages to endocrine disruptors may have increased risk of menstrual cycle irregularities, infertility, endometriosis, autoimmune disorders, and cancers of the reproductive system. Now NIEHS grantee Gayle C. Windham of the Department of Health Services in Oakland, California, and colleagues have found that the pesticide DDT and its metabolite DDE were associated with menstrual length differences in a population of immigrant women from Southeast Asia.

DDT was one of the first chemicals to be shown to have adverse endocrine effects. In wild birds, especially those high on the food chain, DDT was linked with weakened eggshells, which caused large drops in the numbers of some species of raptors including the bald eagle. DDT was shown to interfere with the deposition of calcium as the developing egg passes through the bird’s uterus. For this and other reasons, its use was banned in the United States in 1972.

The California researchers studied 50 Laotian women of reproductive age currently residing in the San Francisco Bay area. The team examined serum samples for suspected endocrine disruptors including DDT, DDE, 4 other chlorinated pesticides, and 10 polychlorinated biphenyls. They found that serum samples from all the women in the study had detectable concentrations of DDT and DDE, with mean levels higher than typical of U.S. women.

Menstrual cycle length was approximately four days shorter for women with the highest DDT and DDE levels compared to women with the lowest levels. With each doubling of serum DDE (though not DDT), cycle length decreased by a little more than one day. Also, as DDE level increased, progesterone metabolite levels decreased. There was no significant association between polychlorinated biphenyl levels and changes in cycle length or hormone levels.

These results indicate an effect of DDT exposure on ovarian function and menstrual cycle length, potentially contributing to problems with fertility, pregnancy, and other aspects of reproduction. The findings need to be duplicated because of the small size of the study population, but they do suggest that DDT exposure may be an important factor in reproductive problems. These human health effects also have implications for the continued use of DDT and similar compounds in other parts of the world.

## Figures and Tables

**Figure:**